# The neutrophil: one cell on many missions or many cells with different agendas?

**DOI:** 10.1007/s00441-017-2780-z

**Published:** 2018-02-12

**Authors:** Gustaf Christoffersson, Mia Phillipson

**Affiliations:** Department of Medical Cell Biology, Uppsala University, Husargatan 3, P.O. Box 571, 751 23 Uppsala, Sweden

**Keywords:** Subpopulations, Marginated pools, Development, Spleen, Lung

## Abstract

The unique role of neutrophils in host defense is not only based on their abilities to kill bacteria but is also due to their abundance in circulation and their ability to quickly migrate and accumulate in great numbers at afflicted sites. The high number of circulating neutrophils is the result of regulated release of new neutrophils from bone marrow as well as from marginated pools to balance their recruitment to tissue. Marginated pools, such as the spleen and lung, have previously been attributed to passively delay neutrophil transit time due to their large capillary network, but recent reports demonstrate that they are comprised of neutrophils with specific functions. The spleen, for instance, holds neutrophil subpopulations at different anatomical locations with distinct functions important for, e.g., bacterial eradication, and the lung was recently shown to re-educate neutrophils that had trafficked from a site of sterile injury to home back to bone marrow for elimination. Further, recent reports demonstrate subpopulations of neutrophils with different actions during homeostasis, infection, tissue restitution and cancer. It is becoming increasingly clear that this cannot be due to different stages of neutrophil activation during their life span but instead points towards distinct subpopulations of neutrophils with different effector functions. Whether these cellular distinctions are due to different education or origin is, however, not yet known. Together, the accumulating information about the heterogeneous neutrophils presents important insights into their role in development of pathologies, as well as revealing novel targets in the form of certain subpopulations to treat disease.

## Introduction

The granulocytic neutrophils are the first cells to be recruited from circulation to the infected tissue. When at the affected site, they are highly efficient in eradicating bacteria by producing reactive oxygen species (ROS), releasing their cytotoxic granular contents, forming extracellular traps and by phagocytosing the invaders. The essential role of neutrophils in the host defense system is evident in persons with neutropenia (e.g., congenital or caused by chemotherapy or infection), which have an increased risk of acquiring opportunistic infections. To do the job, neutrophils rely on their ability to rapidly accumulate in large numbers at the site of infection following recruitment from blood. To allow for this, concomitant neutrophil mobilization from bone marrow and marginal pools to circulation is required to uphold the available circulating population. Another crucial neutrophil feature is the ability to quickly release preformed and granule-stored effector molecules at the afflicted site. The long-term dogma describes neutrophils with very limited protein synthesis potential after maturation, which, together with their short lifespan, protects the host from neutrophil-related collateral damage due to the release of neutrophil granule content at the wrong time and place. However, it is becoming increasingly evident that neutrophils do not constitute a homogenous group of cells but instead are found in different flavors in circulation as well as in tissues. In this review, we will first briefly present neutrophil development and maturation, and then discuss the role of marginated pools for neutrophil trafficking and effector functions, together with the different neutrophil subtypes hitherto described.

## Neutrophil development

Granulopoiesis takes place in the hematopoietic cords of the bone marrow where the committed granulocytic progenitors arise from hematopoietic stem cells and undergo proliferation and differentiation into neutrophils. Fully differentiated and mature neutrophils are then stored in bone marrow and form an extensive reserve to be released upon demand. In the adult human, the bone marrow generates 5–10 × 10^10^ granulocytes per day during steady state (Summers et al. 2010). The primary regulator of the physiological granulopoiesis is the cytokine granulocyte colony-stimulating factor (G-CSF) (Richards et al. 2003), which promotes commitment of progenitor cells to the myeloid lineage, proliferation of granulocytic precursors, and finally the release of neutrophils from the bone marrow through the venous sinusoids. In humans, a dominant negative mutation of the G-CSF receptor causes severe neutropenia (Sinha et al. 2003), a pathology also observed in mice lacking this receptor (Liu et al. 1996). However, interleukin 6 (IL-6), GM-CSF and IL-3 have also been shown to stimulate granulopoiesis, but in a redundant manner (Kopf et al. 1994; Stanley et al. 1994; Nishinakamura et al. 1996).

**Fig. 1:**
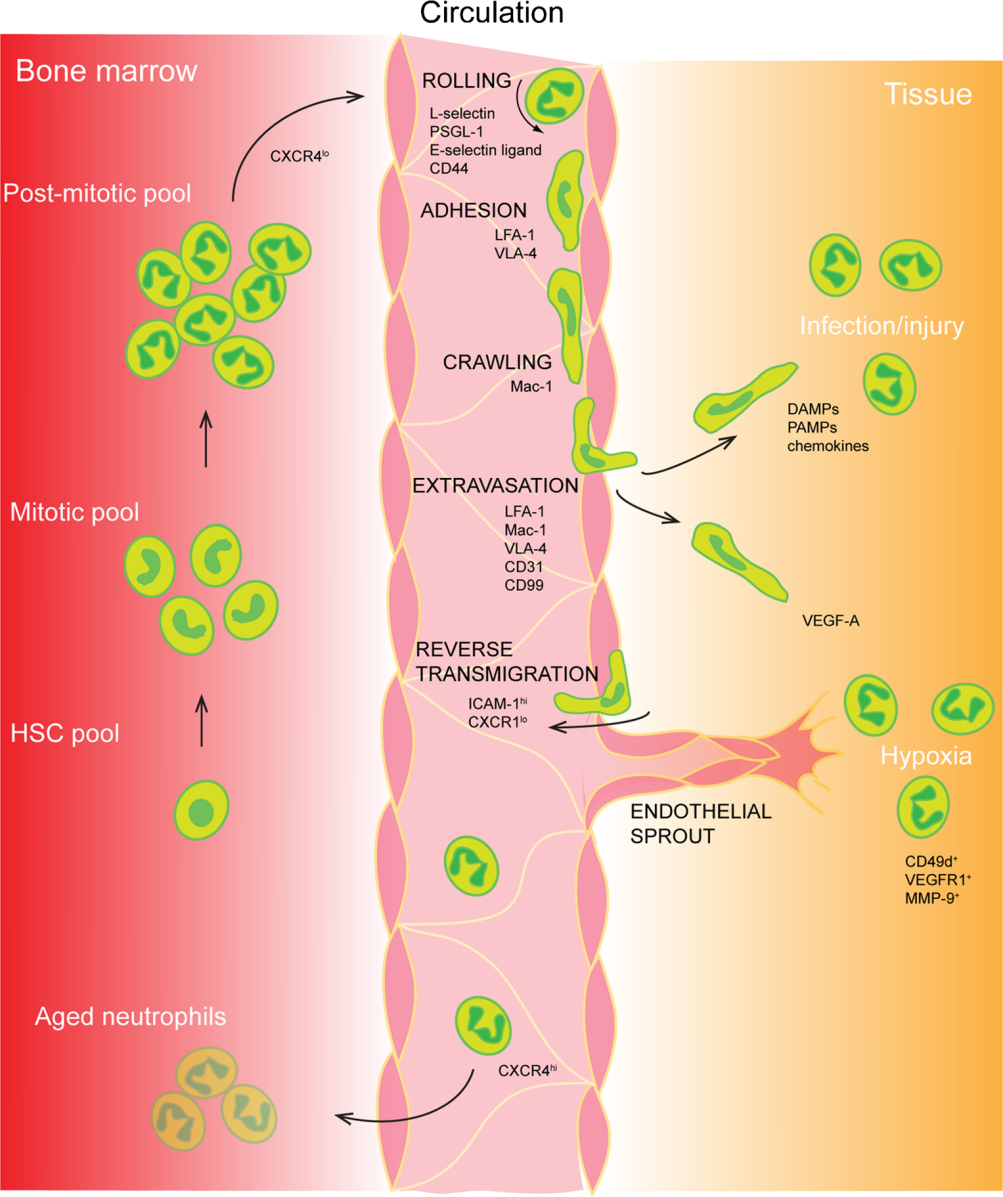
The life of a neutrophil. Neutrophils are generated through granulopoiesis in the bone marrow. They are released into the blood circulation through decreased CXCR4-CXCL12 signaling. Upon injury/infection/hypoxia in tissue, the endothelium will signal to induce the leukocyte recruitment cascade involving rolling, adhesion, crawling, and extravasation. Neutrophils can be recruited to a range of different tissue needs such as infection, sterile injury, and hypoxia. A recently discovered feature of the neutrophil is the ability to leave an injured site to reverse transmigrate into the blood stream to further age and home to the bone marrow to be cleared, also in a CXCR4-CXCL12-dependent manner.* HSC* hematopoietic stem cell,* CXCR* CXC chemokine receptors,* PSGL-1* P-selectin glycoprotein ligand-1,* LFA-1* - lymphocyte function-associated antigen-1,* VLA-4* very late antigen-4,* Mac-1* macrophage-1 antigen,* DAMPs* damage-associated molecular patterns,* PAMPs* pathogen-associated molecular patterns,* VEGF-A* vascular endothelial growth factor-A,* VEGFR1* vascular endothelial growth factor receptor-1,* MMP-9* matrix metalloproteinase-9

Neutrophils exit the bone marrow through the sinusoidal endothelium and thereby enter blood circulation (Campbell 1972). During this process, they maintain the surface level of G-CSFR while down-regulating the chemokine receptor CXCR4 expression (Fig. 1). The ligand to CXCR4, CXCL12/SDF-1 (stromal cell-derived factor 1), is a chemokine expressed under regulation by hypoxia, and is thereby produced by the hypoxic niche in bone marrow (Ceradini et al. 2004). Administration of CXCR4 antagonists induces a rapid increase in circulating neutrophils (Martin et al. 2003; Hendrix et al. 2004), demonstrating that the pathway SDF-1/CXCR4 retains the neutrophils within bone marrow. During inflammatory situations, the release of neutrophils from bone marrow is increased in response to enhanced circulating levels of certain inflammatory mediators such as CXC chemokines (Martin et al. 2003; Burdon et al. 2005).

## Marginated pools of neutrophils

The ability of neutrophils to quickly exit blood circulation to accumulate at affected sites in adequate numbers requires a regulated refilling of the circulating population. While the bone marrow comprises a large reservoir of mature neutrophils, other organs harboring marginated neutrophil pools have been described. The first evidence of the existence of neutrophil marginated pools came in the 1960s, when it was noted that approximately 50% of radioactively labeled neutrophils injected into the circulation of healthy volunteers disappeared from the blood directly after infusion (Mauer et al. 1960). This disappearance of neutrophils was prevented by the addition of adrenaline to the cell infusate, indicating that adrenalin kept the infused neutrophils in the circulation and prevented them from becoming marginated (Athens et al. 1961). Further investigations demonstrated a prolonged neutrophil transit time and thereby the formation of marginated pools of neutrophils in lungs, liver and spleen, in addition to bone marrow. These organs have in common a large capillary network, and the size of a marginated pool depends on both organ blood flow and neutrophil transit time through that specific vascular bed. Whether the marginated pools of the different organs comprise specific clubs for neutrophils of different functions or whether neutrophils were attaining certain functions by education at the site has not been clarified.

The spleen is a secondary lymphoid organ, which plays an important role in the development of an effective immune response via the activation of antibody-producing B lymphocytes. Puga et al. have described two populations of splenic neutrophils exhibiting phenotypes different from circulating neutrophils that were located in the peri-marginal zone and interacting with marginal zone B cells (Puga et al. 2011). These subpopulations (neutrophil B cell-helper 1 and 2, N_BH1_ and N_BH2_) induce T cell-independent B cell activation and elicit class switching, somatic hypermutation and production of immunoglobulins through the release of the factors BAFF, APRIL and IL-21, leading to the generation of an efficient antimicrobial immunoglobulin shield. In addition, two additional distinct subsets of neutrophils (Ly6G^hi^ and Ly6G^int^) populating the red pulp in the spleen were recently identified (Deniset et al. 2017). During infection by *Streptococcus pneumoniae,* the Ly6G^hi^ mature neutrophils scanning the tissue eliminate the bacteria caught at the surface of red pulp macrophages, while the Ly6G^int^ immature neutrophils increase their proliferation rate and convert into resident Ly6G^hi^ neutrophils.

**Figure 2:**
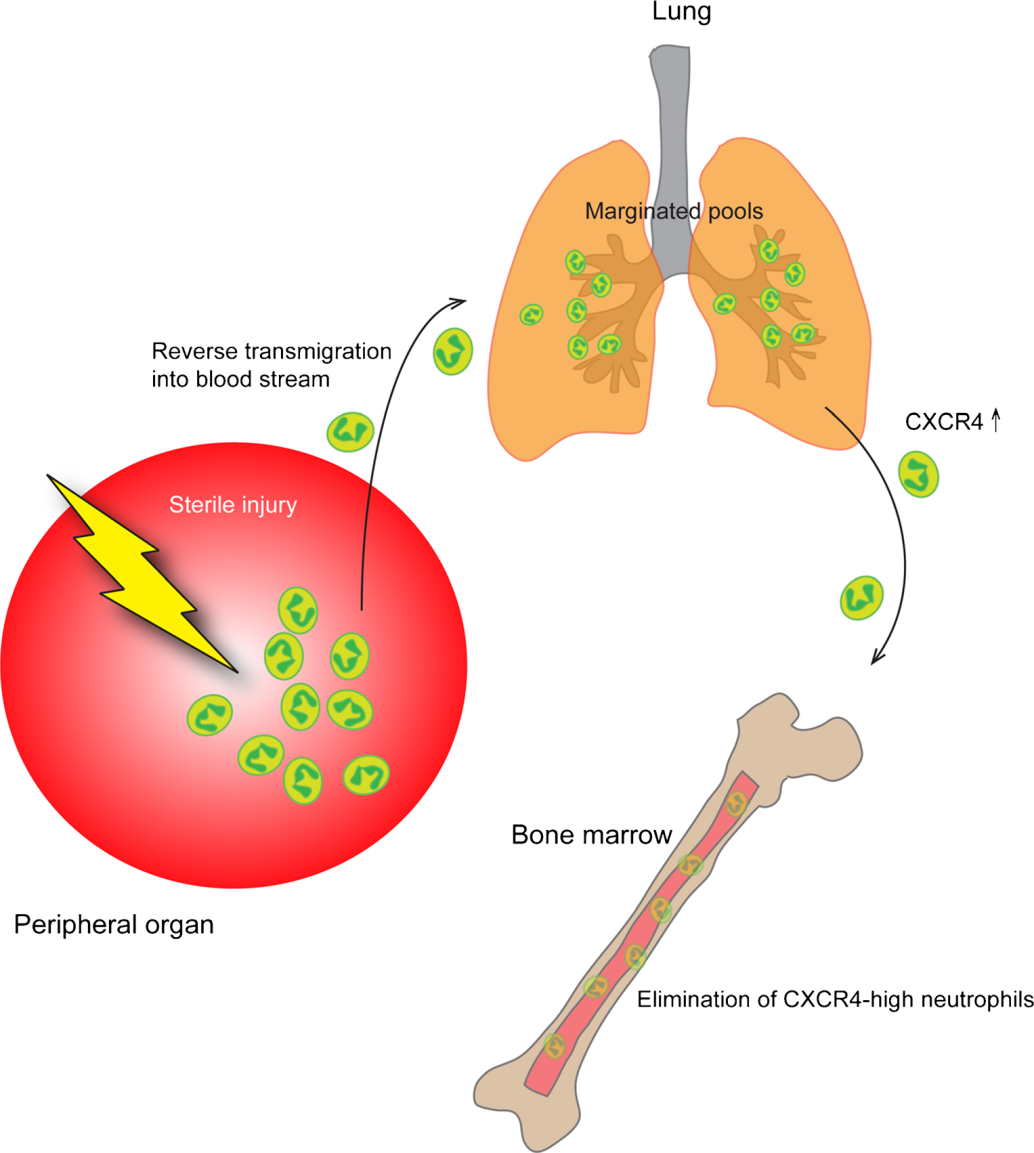
Reverse transmigration and peripheral pools. Neutrophils have recently been found to be able to exit damaged tissue (sterile inflammation or ischemic tissue), and re-enter the blood stream. They thereafter enter marginated pools such as the lung vasculature where they are re-educated to upregulate CXCR4 and later home to the bone marrow for elimination

In the lungs, most marginated neutrophils are found in the alveolar capillaries (Doerschuk et al. 1987), and their delayed transit might depend on neutrophils having a larger diameter than the smallest capillaries and therefore having to deform to go through, but also that neutrophils patrol the lung vasculature in search of microbial invasion (Wang et al. 2017; Yipp et al. 2017). The marginated pool of the lungs has so far been attributed to serve as a reservoir for refilling the circulating population when needed. However, recent data suggest that the marginated neutrophils in lungs become deactivated and primed to traffic to bone marrow to undergo apoptosis (Fig. 2) (Wang et al. 2017). This study demonstrated that neutrophils played an important role in tissue restoration following sterile injury of the liver, as they cleaned up dying cells and allowed for collagen deposition. By photoactivating the neutrophils at the injured site, Wang et al. showed that these cells, after fulfilling their duties in the liver, reverse-transmigrated into nearby blood vessels and trafficked to the lung (Fig. 2). Instead of causing local damage, these neutrophils were found to adhere firmly to the endothelium and to up-regulate the chemokine receptor, CXCR4, prior to trafficking back to bone marrow, where they were cleared.

## Neutrophil subsets

The subsetting of neutrophils is an ongoing matter of discussion; subsetting of other types of immune cells such as T cells and dendritic cells depending on their function, experience, and protein expression has provided immunologists with a plethora of established populations of specialized cells. For neutrophils, however, defining bona fide subsets has proven to be more difficult and has to overcome two well-accepted assumptions about these cells: (1) their short lifespan (a few hours in circulation) (Dancey et al. 1976; Suratt et al. 2001), and (2) their limited protein synthesis machinery that restricts their ability to change phenotype following granulopoiesis to adapt to new challenges or microenvironments (Cline 1966; Klebanoff and Clark 1978; Hughes et al. 1987). In addition, confounding factors in defining different subsets of neutrophils are associated with their ability to change phenotype in the extra-marrow maturation process, as well as with activation. It has now been demonstrated that activation of neutrophils by cytokines (e.g., TNFα, IL-1β, IL-17) and bacterial compounds (e.g., LPS, fMLP) greatly prolongs their lifespan to up to 5 days (Pillay et al. 2010), as well as inducing phenotype changes (Theilgaard-Monch et al. 2004; Chakravarti et al. 2009; Fortunati et al. 2009). During the last years, we and others have reported the identification of distinct neutrophil subtypes in mouse and human circulation during homeostasis (listed in Table 1). In addition, other subtypes are found during specific disease conditions, such as cancer and sepsis. However, whether these subpopulations are generated per se in bone marrow during granulopoiesis or the result of a later education/adaptation still remains unknown.

**Table 1:** The features and functions of neutrophil subtypes

Neutrophil subtype	Defining features	Function	Model system	Reference
N1 (antitumor)	Hypersegmented nuclei, high TNFα, ICAM-1, FAS	Cytotoxic (through ROS)	Mouse, subcutaneous and orthotopic tumors	Fridlender et al. 2009
N2 (protumor)	Circular nuclei, high arginase, CCL2, CCL5	T cell inactivation, angiogenic factors, matrix degradation		
N1 (pro-inflammatory)	Ly6G^+^, CD206^−^	Promotes adverse ventricle wall remodeling	Mouse, myocardial infarction	Ma et al. 2016
N2 (anti-inflammatory)	Ly6G^+^, CD206^+^	Attenuates adverse ventricle wall remodeling		
SiglecF^hi^ (protumor)	SiglecF^hi^,	Cancer promotion; myeloid cell recruitment, angiogenesis, T cell suppression	Mouse, KP lung tumor, Human, non-small cell lung cancer patients	Engblom et al. 2017
Pro-tumorigenic	VEGF, MMP-9, CXCR4, IFNγ-responsive	Proangiogenic, matrix degradation	Mouse, subcutaneous tumors and metastases, Matrigel assay	Jablonska et al. 2010
Pro-angiogenic	CD49d, CXCR4, VEGFR1, MMP-9	Accumulate at hypoxic sites and stimulate angiogenesis	Mouse, transplanted hypoxic tissue, hindlimb ischemia, recruitment models	Christoffersson et al. 2012, Massena et al. 2015
Reverse-migrated	Activated morphology	Can re-mount responses to microbes and injuries	Zebrafish wounding and infection	Ellett et al. 2015
PMN-N	“Normal” neutrophils, round nuclei, CD49d^−^, CD11b^−^, TLR-2,-4,-9	No effect on macrophages	Mouse, MRSA infection	Tsuda et al. 2004
PMN-I	Multilobular nuclei, CD49d^+^, CD11b^−^, IL-12, CCL3, TLR-2,-4,-5,-8	Activates M1 macrophages		
PMN-II	Ring-shaped nuclei, CD49d^−^, CD11b^+^, IL-10, CCL2, TLR-2,-4,-7,-9	Activates M2 macrophages		
IL-17A-producing neutrophils	RORγt, IL-17	Clear infections	Human blood, cystic fibrosis – pseudomonas aeruginosa-infected, mouse, fungal infection	Taylor et al. 2014, 2016,
High density neutrophils	Hypersegmented nuclei	Anti-tumor	Mouse, tumor models, Human cancer	Sagiv et al. 2015
Low density neutrophils	Banded/ring-shaped to hypersegmented nuclei	Tumor-permissive		
CD62L^bright^/CD16^bright^	Multilobular nuclei,		Human; LPS administration to volunteers	Pillay et al. 2010, 2012
CD62L^dim^/CD16^bright^	Hypersegmented nuclei, CD11c^hi^, CD11b^hi^, CD54^hi^	ROS release to control T cell proliferation		
CD62L^bright^/CD16^dim^	Banded nuclei, CD11c^lo^, CD11b^lo^, CD54^lo^			

A few neutrophil subsets have been described to be constitutively present in the blood stream. One of the most studied subset is defined by the expression of the GPI-anchored protein CD177 on the cell surface, a receptor for proteinase-3 (PR3) (Li et al. 2015). The function of the complex CD177–PR3 is still poorly understood, but a recent study shows a potential role as a modulator of neutrophil recruitment from the circulation, as ligation of CD177 promotes immobilization of neutrophils during transmigration in association with β2-integrin signaling (Bai et al. 2017). The CD177–proteinase-3 complex (mPR3) gained increased attention when it was discovered that autoantibodies targeting this complex were found in patients with anti-neutrophil cytoplasmic antibody-dependent vasculitis (Jerke et al. 2011). Increased levels of this autoantibody correlate with relapses, but the functional importance of the CD177^+^ neutrophils in this disease (and in health) is still unclear.

Another recently identified neutrophil population marker found in human is olfactomedin-4 (OLFM4) which is expressed in neutrophil-specific granules of some mature neutrophils (Clemmensen et al. 2012). As with CD177, the function of OLFM4 is unknown, but mouse studies suggest that this protein may inhibit cathepsin C, and can thereby influence the neutrophil’s capacity to kill bacteria. In several mouse models deficient of OLFM4, the ability to kill bacteria was reduced (Liu et al. 2010, 2012, 2013).

An intriguing observation was the discovery of a subset of neutrophils (about 5–8% of the population) expressing T cell receptors (TCRs) αβ at their cell surface (Puellmann et al. 2006), otherwise found mostly on T lymphocytes, as well as the RAG1/RAG2 recombinase complex. By using nude mice, which are deficient in T cells, the authors demonstrated that this expression was not simply due to neutrophil engulfment of T lymphocytes. Even though the reason for the neutrophils to express this receptor is unclear, the ligation of the TCR complex resulted in protection of neutrophils from apoptosis and stimulation of IL-8 secretion (Puellmann et al. 2006).

## In infection

Neutrophils have traditionally been recognized as first responders to infections, where they rapidly extravasate from the vasculature into tissue to fight intruding microorganisms. However, using different models of infection, it is becoming increasingly clear that not all neutrophils are on the same mission.

In an experimental murine model of infection by methicillin-resistant *Staphylococcus aureus* (MRSA), Tsuda and colleagues isolated two subsets of neutrophils (PMN-I and PMN-II), which differ from those obtained from naive mice (PMN-N, normal neutrophils) (Tsuda et al. 2004). PMN-I were isolated from MRSA-resistant hosts, expressed the toll-like receptors (TLR) -2, -4, -5 and -8, released IL-12 and the chemokine ligand CCL-3, and activated classically-activated M1 macrophages. On the other hand, PMN-II were from MRSA-sensitive hosts, expressed the TLR-2, -4, -7 and -9 and activated alternatively-activated M2 macrophages. However, how these subsets are induced is not yet completely clear.

Interleukin 17A (IL-17) is a cytokine involved in the development of many autoimmune diseases as well as in inflammatory conditions. It is produced by cells expressing the transcription factor RORγt, which comprise T helper cells (T_H_17), natural killer T cells, γδ T cells, and innate lymphoid cells. However, neutrophils have also been identified as an important source of IL-17 in response to LPS-induced lung inflammation and *Aspergillus fumigatus* infection in mice and humans (Ferretti et al. 2003; Werner et al. 2011). Recently, several studies described a subset of neutrophils, which when stimulated by the cytokines IL-6 and IL-23, express the receptor IL-17R and produces the cytokine IL-17A under the control of the transcription factor RORγt (Taylor et al. 2014, 2016). IL-17 displays an autocrine activity on this neutrophil population, which induced an enhanced production of ROS and thereby increased the killing of invading fungi.

Neutrophils are the first responders to infections, where they rapidly extravasate from the vasculature into tissue to fight intruding microorganisms. An ongoing infection will promote neutrophilia, which may lead to excessive inflammation and tissue damage, thus neutrophil recruitment and action has to be tightly regulated. Recent reports describe a neutrophil subset producing the immunosuppressive cytokines IL-10 in response to bacterial and fungal infections. In acute mycobacterial infection, the inflammatory response of monocytes and macrophages is damped by the secretion of large amounts of IL-10 from neutrophils (Zhang et al. 2009), a phenomenon also observed in a model of septic peritonitis where adoptive transfer of WT but not IL-10^−/−^ neutrophils decreases the release of TNF by monocytes (Ocuin et al. 2011). Moreover, in a model of *Trypanosoma cruzi* infection, a dual role of neutrophils was highlighted: the neutrophils first displayed an inflammatory response against the parasite, and then, after activation by IL-17A, migrated to the spleen and liver to release IL-10, leading to decreased IFN-γ production and tissue damage (Tosello Boari et al. 2012). While it has been demonstrated that a cell–cell contact with LPS-activated regulatory T cells triggers the IL-10 production by neutrophils (Lewkowicz et al. 2016), the mechanism is still poorly understood as well as if only a subset of neutrophils can display this immunosuppressive feature.

However, in a model of simulated sepsis in humans (LPS injection in healthy volunteers), an anti-inflammatory subset of neutrophils that surfaced during late stages of simulated sepsis was detected (Pillay et al. 2010). In this, and in a follow-up study, the authors found a subset of mature neutrophils with hypersegmented nuclei defined as CD16^bright^CD62L^dim^ which suppressed T cell activation through release of ROS (Pillay et al. 2012). Along with this subset, CD16^bright^CD62L^bright^ neutrophils with multilobular nuclei, and newly-released CD16^dim^CD62L^bright^ with banded nuclei, were consistently found in the circulation of the LPS-challenged individuals.

Thus, heterogeneity within the neutrophil population during infection has been demonstrated in multiple scenarios, and different subsets have clearly defined roles in promoting and suppressing the inflammatory response.

## In tissue restitution and healing

Following the inflammatory phase, neutrophils also participate in tissue remodeling during the restitution phase. Tissue hypoxia due to myocardial infarction was shown to first recruit pro-inflammatory neutrophils (“N1”), with high expression of e.g. *Il-1β, Il-6, and Tnf-α,* but in later stages (days 5–7), anti-inflammatory (“N2”) neutrophils emerged with the expression of, e.g., *Arg1, Il-10,* and *Ym1* (Ma et al. 2016). The N2 were found to be Ly6G^+^ CD206^+^ and were activated in the post-ischemic tissue, and likely added to healing of the myocardium. The N1 cells were, on the other hand, involved in ventricular wall thinning when lingering in the tissue, because of their release of proteases. This was the first time a stark division of neutrophil polarization was found in an ischemic model.

Our group recently described a distinct neutrophil subtype that was recruited by vascular endothelial growth factor (VEGF)-A to sites of tissue hypoxia, where they display a pro-angiogenic phenotype (Massena et al. 2015). This subpopulation constitutes about 3–5% of the circulating neutrophils of both humans and mice, and, in contrast to other neutrophils, they express a high level of the VEGF-A receptor VEGFR1. Further, we demonstrated the essential role of the integrin VLA-4 (CD49d/CD29, α4/β1) expressed by the pro-angiogenic neutrophils, for their recruitment from blood circulation to the site of hypoxia. We have also found that the pro-angiogenic neutrophils display higher levels of the chemokine receptor CXCR4 and content of the matrix metalloproteinase (MMP)-9 compared to neutrophils recruited to inflammatory sites. After being recruited to hypoxic areas, they interact with sprouting endothelium (Christoffersson et al. 2017), facilitating vessel growth through the release of matrix metalloproteinase-9 (MMP-9) (Christoffersson et al. 2012).

## In cancer

The involvement of neutrophils in both prevention and promotion of tumor progression has been suggested for several decades, and contradictory evidence has fuelled the controversy about the role of neutrophils in cancers. A confounding cell type when studying neutrophils in tumors is the myeloid-derived suppressor cell (MDSC), which shares morphological features with neutrophils and the expression of the surface marker Gr-1. These MDSC exerts a ROS- and arginase1-dependent immunosuppressive function on T cell proliferation and activation, and thereby limit the endogenous anti-tumor response (Marvel and Gabrilovich 2015). Thus, most defining features of MDSCs are functional features and intracellular proteins making the immediate distinction from neutrophils difficult.

In their seminal study, Fridlender and colleagues showed that neutrophils in tumors could be divided into two separate subsets based on their pro- and anti-tumor functions, as well as their expression and morphology (Fridlender et al. 2009). They named them N1 and N2 with reference to the more established division of macrophages into the classically activated ‘M1’, and the alternatively activated ‘M2’ (Mills and Ley 2014), and demonstrated that the polarization to the anti-tumoral N1 or the pro-tumoral N2 phenotype is driven by the cytokine interferon gamma (IFN-γ) and transforming growth factor beta (TGF-β), respectively (Jablonska et al. 2010; Andzinski et al. 2016). Anti-tumoral tumor-associated neutrophils (TANs) N1 are characterized by high production of pro-inflammatory cytokines, an hypersegmented nuclei, and high tumor-cell-killing abilities, whereas the N2 subtype is characterized by an increased arginase content, an immature morphology, and displays tumor-promoting function (Fridlender et al. 2009). The transcriptomic analysis of these subsets compared to MDSC and naive neutrophils (isolated from tumor-free mice) showed that they were indeed three distinct subsets (Fridlender et al. 2012), but definitive evidence of a possible common progenitor is still lacking. However, in recent studies about low- and high-density granulocytes (LDG and HDG, respectively), it has been demonstrated that mature suppressive neutrophils in the LDG fraction originate from the HDG fraction (Sagiv et al. 2015). In addition, these HDG neutrophils acquired their suppressive functions following stimulation with TGF-β and may explain the origin of the TAN N2 previously described. The authors also suggested that immature LDG neutrophils are released from the bone marrow before the termination of the maturation steps. On the other hand, the possibility that LDG neutrophils acquired their density phenotype and suppressive functions in parallel processes is supported by LDGs isolated from systemic lupus erythematosus patients which do not display immunosuppressive features (Denny et al. 2010). Intriguingly, results from in vitro experiments showed that degranulation of circulating neutrophils could be a prerequisite to their acquisition of the low-density and immunosuppressive features (Rodriguez et al. 2009), suggesting that neutrophils may go through several specialized phenotypes during their lifespan.

The ability of solid tumors to affect hematopoiesis and thereby the fate and phenotype of circulating neutrophils was recently demonstrated (Engblom et al. 2017). This study shows that lung adenocarcinomas activate osteoblasts at distant sites, which drive tumor progression by supplying them with pro-tumorogenic neutrophils expressing high levels of Siglec-F (sialic acid-binding immunoglobulin-like lectin F). These specific neutrophils attained most of their protumor-effects at the tumor site, but higher levels of Siglec-F was found on circulating neutrophils in tumor-bearing mice, and correlated with worse outcome in patients with lung adenocarcinomas. The mechanisms by which the tumor directly affect hematopoiesis remain to be full uncovered, but seems to involve increased levels of circulating RAGE (receptor for advanced glycation end products).

## Concluding remark: maturation stages or true subsets?

It is evident that neutrophil functions go well beyond those of the kamikaze cells predestined to find and kill bacteria before dying at the site of infection. Many studies have demonstrated that apart from their important role in host defense, neutrophils are also crucial actors in angiogenesis, tissue restoration and resolution of inflammation. In addition, new studies reveal specific functions of the marginated pools that reside in distinct organs with large vascular beds, and thereby enabling vast neutrophil–endothelial cell interactions or even microenvironmental instructions. However, further investigations, such as sequencing of the transcriptomes of the defined neutrophil subpopulations, will uncover their individual toolboxes and potential fate. In addition, lineage-tracing experiments are needed to unequivocally answer the question whether these various functions are performed by neutrophil populations of distinct origin or by neutrophils at different levels of maturations or with different education.
